# Supplementary figures and images for: Aggregation chimeras provide evidence of in vivo intercellular correction in ovine CLN6 neuronal ceroid lipofuscinosis (Batten disease)
Source: PLoS One. 2022 Apr 11;17(4):e0261544. doi: 10.1371/journal.pone.0261544 (PMC9000108; doi:10.1371/journal.pone.0261544)

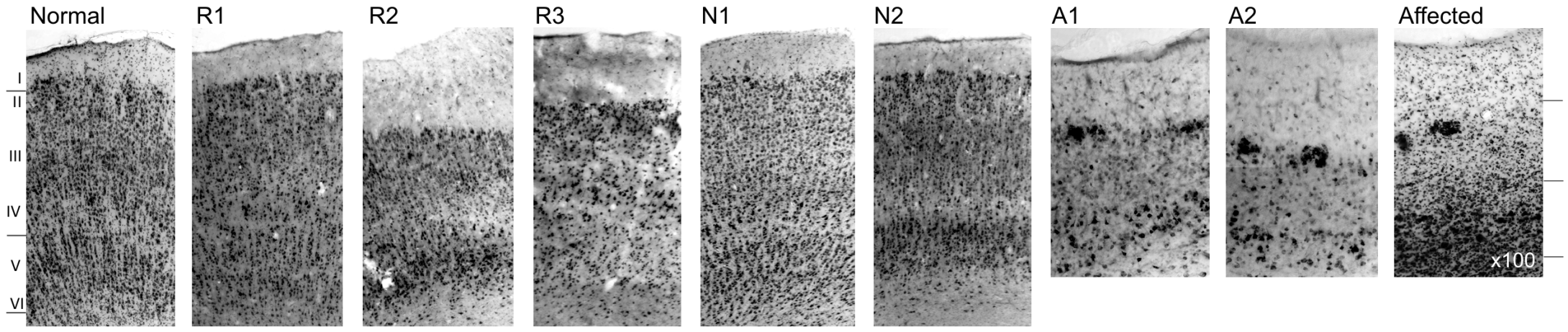

Supplement: S1 Fig — (TIF) [file pone.0261544.s001.tif]

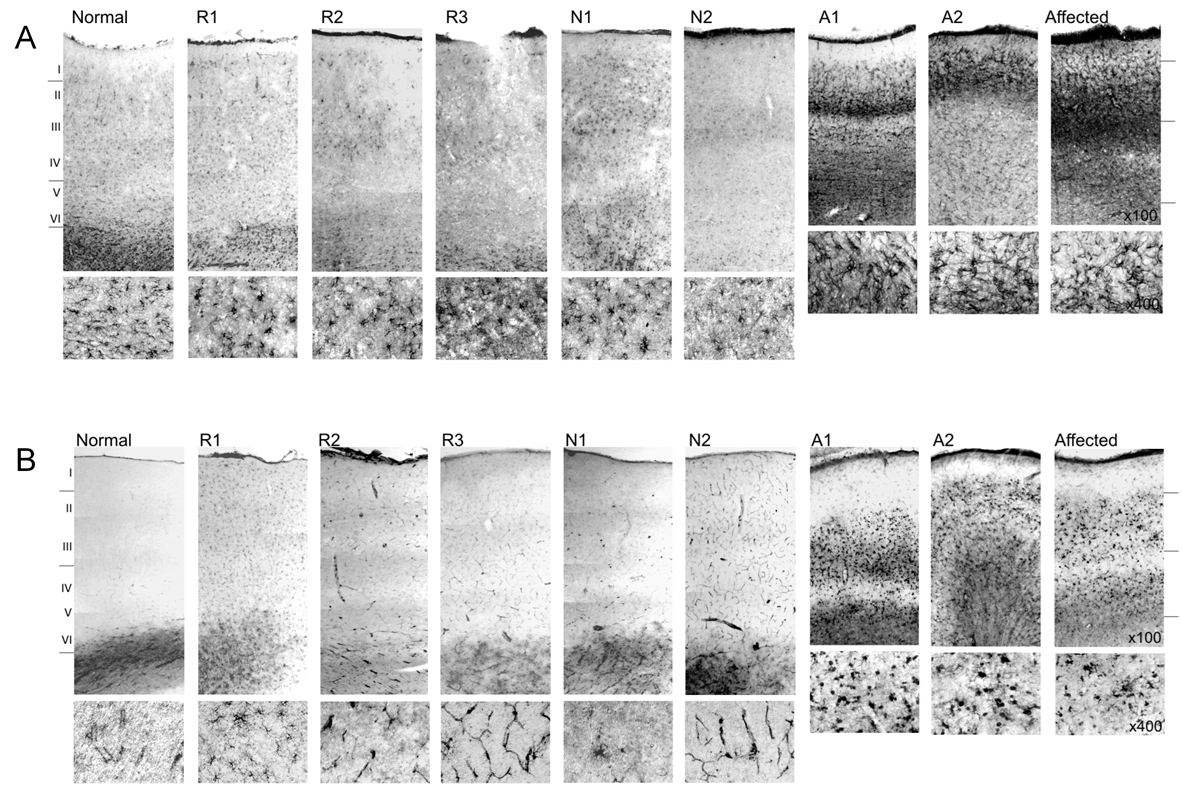

Supplement: S2 Fig — A) GFAP astrocytic staining and B) GSB4 microglial staining animals of normal, affected and chimeric occipital cortex. (TIF) [file pone.0261544.s002.tif]

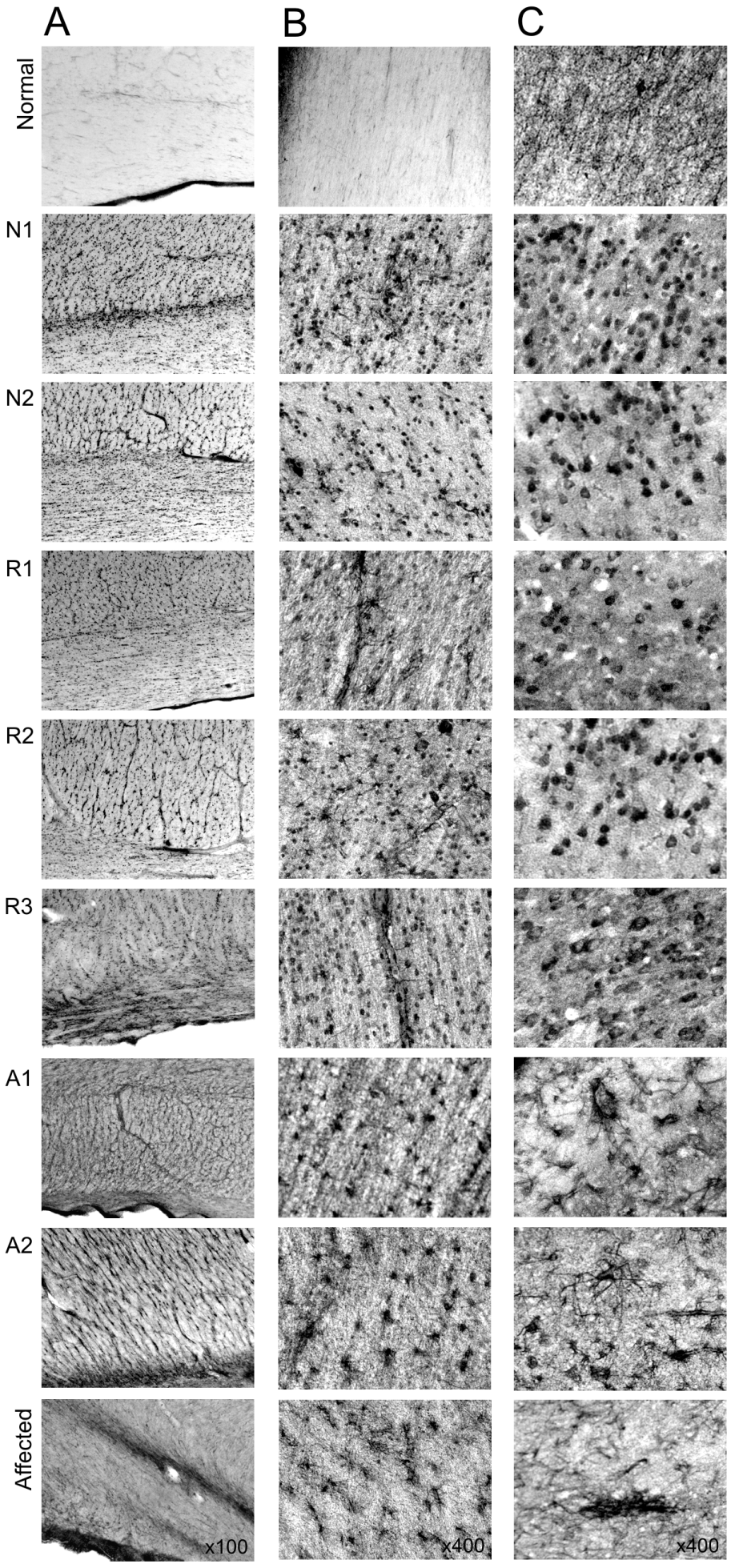

Supplement: S3 Fig — PSA-NCAM staining along the SVZ (A) and within the white (B) and grey matter (C) of control and chimeric animals. (TIF) [file pone.0261544.s003.tif]
