# Supplementary material for: Aggregation chimeras provide evidence of in vivo intercellular correction in ovine CLN6 neuronal ceroid lipofuscinosis (Batten disease)
Source: PLoS One. 2022 Apr 11;17(4):e0261544. doi: 10.1371/journal.pone.0261544 (PMC9000108; doi:10.1371/journal.pone.0261544)

S1 - Raw Gel images

The following raw gel photographs were used to generate **Fig. 2 Restriction enzyme detection of the c.822G>A polymorphism to determine the extent of chimerism**. Digested PCR products were separated on 4% agarose gels and images captured with a Gel Doc XR+ gel documentation system using Image Lab software (Biorad, CA, USA).

Please note there was some rearranging/splicing of the lanes from these raw images (as indicated) in the final image to ensure matching of regions between animals.

The following raw gel image was used to generate **Fig. 2A**. A 277bp PCR product from normal **(GG)** sheep cleaved with *HaeII* results in three bands of 119, 91 and 67bp, affected **(AA)** sheep yield two bands of 186 and 91bp, and heterozygous **(GA)** sheep four bands of 186, 119, 91 and 67bp. This gel image shows serial dilutions of affected and normal DNA which were used as a standard to estimate the proportion of affected DNA present in samples from chimeric animals by visual inspection. Numbers indicate the affected portion (A %). Please note the presence of a uniform band at 277bp, indicating not all of the 277bp amplicon was digested.

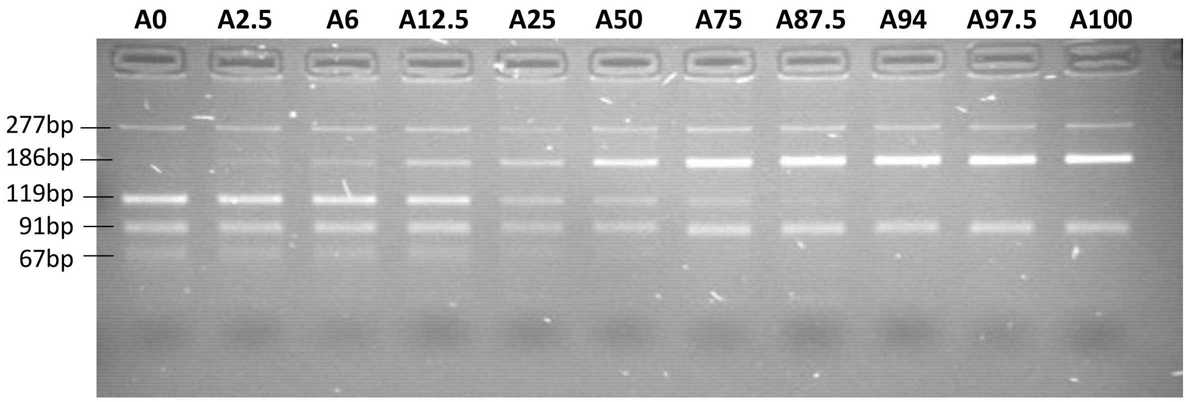

The following five raw gel images were used to generate **Fig. 2B**. The first raw gel shows the PCR products from brain and peripheral tissues from normal-like animal N1. Some lanes were spliced in the manuscript to to ensure matching of regions (brain and peripherals) between animals.

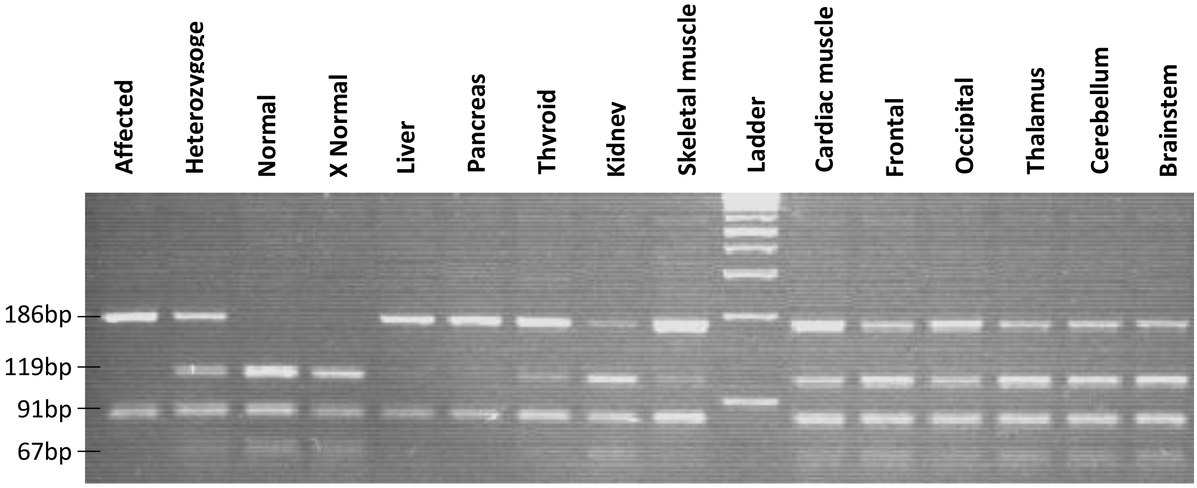

The next two raw gels shows the PCR products from brain and peripheral tissues from recovering-like animal R3.

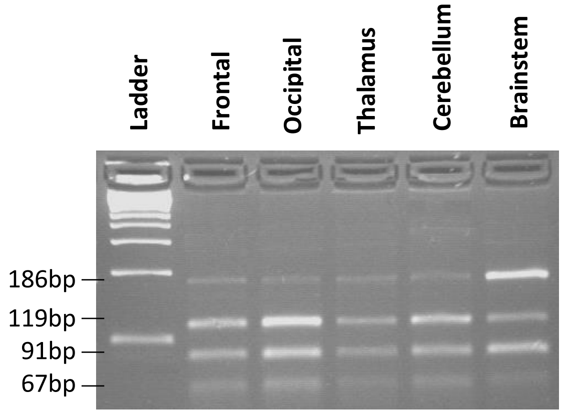

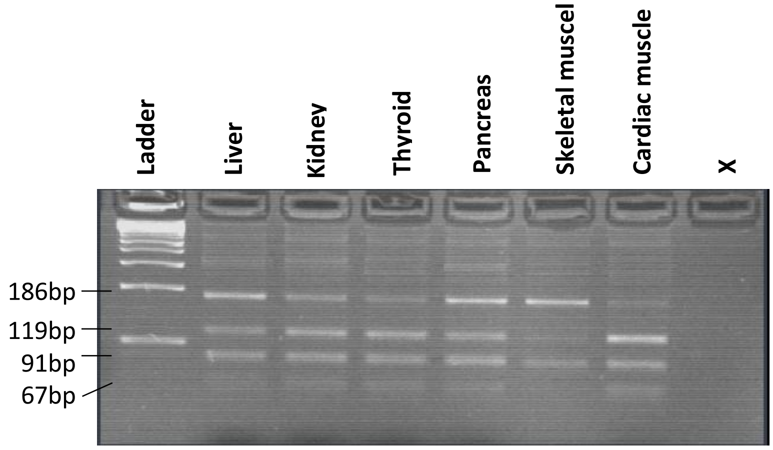

The final two raw gels shows the PCR products from brain and peripheral tissues from affected-like animal A2. Some lanes were spliced in the manuscript to to ensure matching of regions (brain and peripherals) between animals. Please note the presence of a uniform band at 277bp in the first gel, indicating not all of the 277bp amplicon was digested.

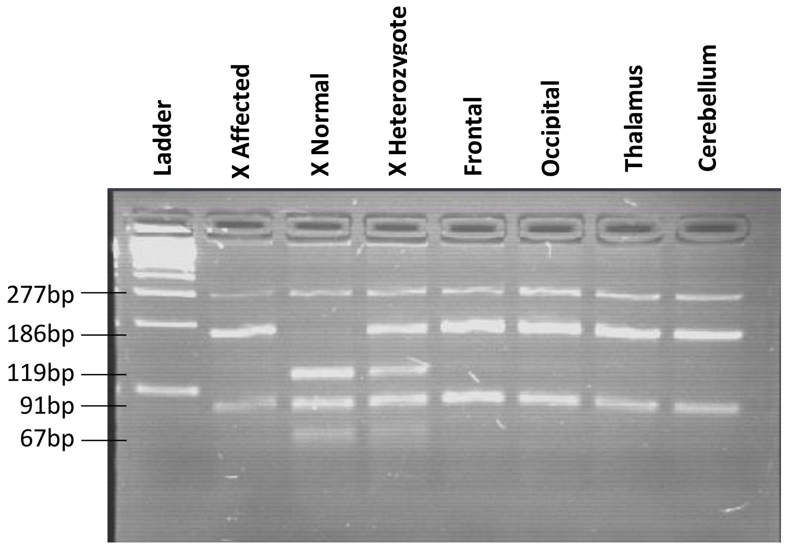

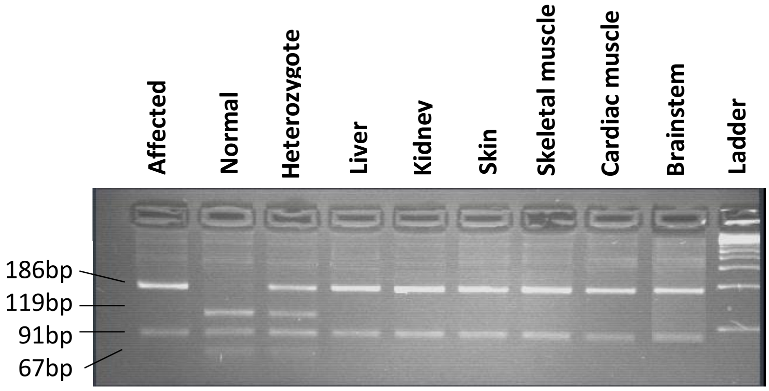

Supplement: S1 Raw images — (PDF) [file pone.0261544.s004.pdf]
